# Cell Wall Calcium and Hemicellulose Have a Role in the Fruit Firmness during Storage of Blueberry (*Vaccinium* spp.)

**DOI:** 10.3390/plants10030553

**Published:** 2021-03-16

**Authors:** Patricio Olmedo, Baltasar Zepeda, Bárbara Rojas, Christian Silva-Sanzana, Joaquín Delgado-Rioseco, Kamila Fernández, Iván Balic, César Arriagada, Adrián A. Moreno, Bruno G. Defilippi, Reinaldo Campos-Vargas

**Affiliations:** 1 Centro de Biotecnología Vegetal, Facultad de Ciencias de la Vida, Universidad Andrés Bello, 8370186 Santiago, Chile; pa.olmedo@gmail.com (P.O.); biobarbara.rsm@gmail.com (B.R.); c.silva.sanzana@gmail.com (C.S.-S.); joaquin.delgado.rioseco@gmail.com (J.D.-R.); kamila.fernandez.c@gmail.com (K.F.); adrian.moreno@unab.cl (A.A.M.); 2 Horticulture and Product Physiology, Department of Plant Sciences, Wageningen University, 6700 AA Wageningen, The Netherlands; baltasar.zepedapuiggros@wur.nl; 3 Departamento de Acuicultura y Recursos Agroalimentarios, Universidad de Los Lagos, 5310887 Osorno, Chile; ivan.balic@ulagos.cl; 4 Laboratorio Biorremediación, Departamento de Ciencias Forestales, Facultad de Ciencias Agropecuarias y Forestales, Universidad de la Frontera, 4811230 Temuco, Chile; cesar.arriagada@ufrontera.cl; 5 Instituto de Investigaciones Agropecuarias, INIA la Platina, 8831314 Santiago, Chile; 6 Centro de Estudios Postcosecha, Facultad de Ciencias Agronómicas, Universidad de Chile, 8831314 Santiago, Chile

**Keywords:** fruit, texture, xyloglucan, calcium

## Abstract

The firmness of blueberry is one of its most significant quality attributes. Modifications in the composition of the cell wall have been associated with changes in the fruit firmness. In this work, cell wall components and calcium concentration in two blueberry cultivars with contrasting firmness phenotypes were evaluated at harvest and 30 days cold storage (0 °C). High performance anion-exchange chromatography with pulse amperometric detector (HPAEC-PAD) analysis was performed using the “Emerald” (firmer) and “Jewel” (softer) blueberry cultivars, showing increased glucose in the firmer cultivar after cold storage. Moreover, the LM15 antibody, which recognizes xyloglucan domains, displayed an increased signal in the Emerald cultivar after 30 d cold storage. Additionally, the antibody 2F4, recognizing a homogalacturonan calcium-binding domain, showed a greater signal in the firmer Emerald blueberries, which correlates with a higher calcium concentration in the cell wall. These findings suggest that xyloglucan metabolism and a higher concentration of cell wall calcium influenced the firmness of the blueberry fruit. These results open new perspectives regarding the role of cell wall components as xyloglucans and calcium in blueberry firmness.

## 1. Introduction

Blueberries (*Vaccinium* spp.) have grown in importance for consumers since they began being marketed as a superfruit with potent health benefits [1,2,3,4]. This has increased the worldwide production and cultivation of different varieties of blueberries. However, like many other fruits, the quality of the blueberry is a leading consideration for the consumer, one of the most important indicators of which is firmness [5,6,7]. Fruit firmness changes continuously during development and ripening, showing a prominent reduction in firmness after harvest. A great variability has been found in fruit firmness among varieties, even within the same harvest [8,9,10]. Different factors have been proposed to mediate the softening rate in blueberries, including maturation stage at harvest [11], water loss [12], and fruit orientation within the bunch [13], among others. Several studies have pointed out that the processes that underlie changes in blueberry firmness have been mainly related to the disassembly of cell wall components combined with an increase in cell wall-degrading enzymatic activities [14,15,16].

The cell wall is a dynamic and highly complex polysaccharide matrix composed of cellulose, hemicellulose, and pectins. Cellulose fibers are very stable structures; they are organized in linear chains of glucose that interact with each other through hydrogen bonds [17]. On the other hand, hemicelluloses are composed of linear chains of glucose, xylose and/or mannose, and different branches containing these sugars or others, such as fucose and arabinose (Ara), that interact with the cellulose fibers via hydrogen bonds [18,19,20]. Cellulose and hemicellulose are embedded in a matrix of pectins, the latter being the most complex component of the cell wall [21]. Pectins are linear or branched chains of sugars able to interact with each other or with other components of the cell wall [18]. Structurally, pectins comprise three main polysaccharides, namely, homogalacturonan (HG), rhamnogalacturonan-I (RG-I), and RG-II [22]. The role of calcium in fruit ripening processes, its interaction with the cell membrane and the cell wall, and development of physiological disorders has been studied for a long time [23,24,25,26,27]. There are varied publications describing the effect of calcium on the fruit quality of different species such as apple [28], pear [29], cherry [30], grape [31,32], peach [33], and berries [34,35], among others. In blueberry, the use of different compounds containing calcium has been studied in soil applications, foliar and solution dips [36,37,38,39,40,41,42,43,44]. The results show generally positive effects on fruit characteristics in terms of firmness at harvest and after refrigerated storage [14,44,45,46]. In plant models it has been determined in different investigations that calcium modifies the physical properties of the cell wall by interacting with pectins, in particular with nonesterified zones of homogalacturonans, generating a conformation called the egg box [47]. Studies in blueberries have indicated that the differences in texture among the varieties would correlate with differences in the cell wall components [15,48]. Likewise, by analyzing the changes that occur in the cell wall of developing and ripening blueberry fruits, it has been determined that hemicellulose-type components undergo significant changes that would impact the physical properties of the fruit [49]. In addition, calcium applications to blueberry fruit in preharvest have been studied, resulting in a decrease in the rate of softening that correlates with a greater amount of calcium in the cell wall of these fruits [14], which would produce a lower solubilization of pectins [49]. There is little work on characterizing the changes in the cell wall of blueberries postharvest. An increase in the firmness of blueberry fruits in the first days of refrigerated storage has been described, which would be due to an increase in chelator soluble pectin in this period [50], and the decrease in firmness in prolonged storage would correlate with a disassembling of the different components of the cell wall [16].

The loss of blueberry firmness during storage is one of the most important obstacles that must be overcome to prevent postharvest losses. Because this phenomenon has been linked to cell wall degradation [6,16,51], this study aims to evaluate the modifications that occur in the cell wall of the blueberry fruit with contrasting firmness to gain further insights to help solve this intractable quality loss problem.

## 2. Results

### 2.1. Phenotype and Firmness Properties of Blueberry Cultivars at Harvest and after Cold Storage

**Figure 1:**
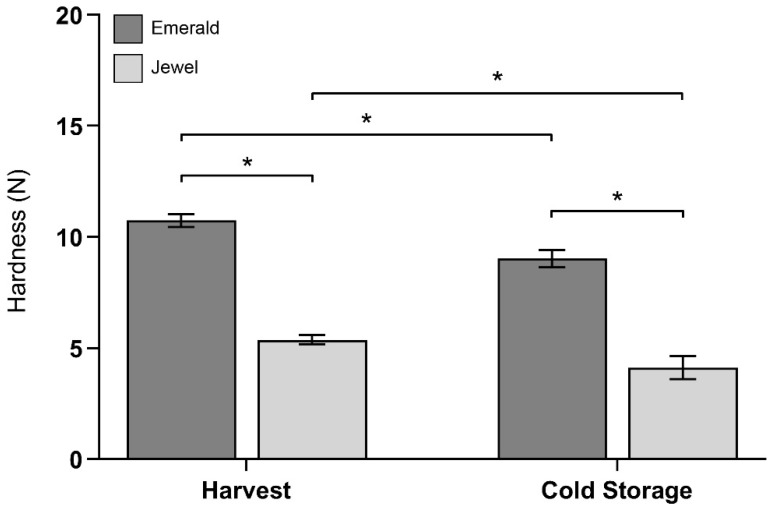
Firmness (as hardness) of two selected blueberry cultivars “Emerald” and “Jewel” at harvest stage and after 30 d of cold storage. Error bars represent SEM (n = 10). Data were analyzed by *t*-test (* *p* < 0.05).

To better understand the differences in the phenotypic features of blueberry cultivars cultivated in the northern region of Chile, the fruit weight, diameter, total soluble solids (TSS), titratable acidity (TA) and hardness of two cultivars (i.e., Emerald and Jewel) were determined at harvest (Table S1). Texture profile analysis (TPA) is composed of several parameters, with hardness being considered a suitable indicator of blueberry firmness [6]. The Emerald and Jewel cultivars exhibited similar weights but contrasting levels of hardness. The Emerald cultivar showed a higher hardness (firmness) compared to Jewel both at harvest and after 30 d of cold storage (Figure 1); the hardness levels were 2.0- and 2.2-fold higher, respectively. Additionally, during 30 d of cold storage, the Jewel cultivar showed a higher firmness loss, being 23.4%, while the Emerald cultivar showed a firmness loss of 15.9%. Based on these observations, the comparison of the cell wall composition of these two cultivars could provide valuable information on the disparities in firmness at different time points.

### 2.2. Global Cell Wall Noncellulosic Monosaccharide Composition Analysis of Emerald and Jewel Blueberry Cultivars by HPAEC-PAD

**Figure 2:**
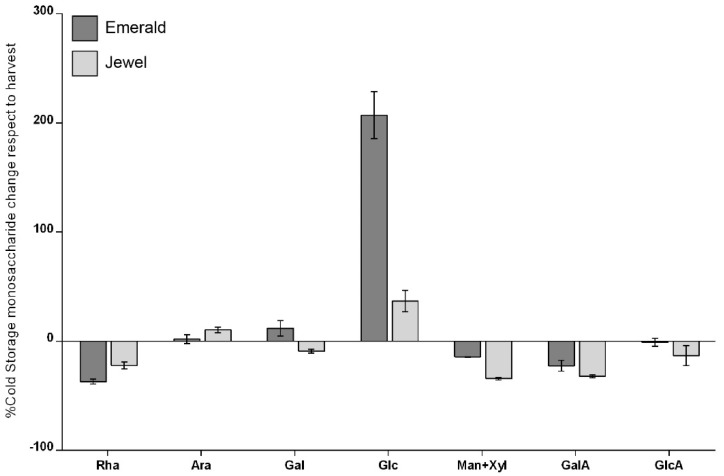
Changes in cold storage stage of cell wall monosaccharide composition percentage with respect to harvest stage. Error bars represent SEM (n = 6).

The monosaccharide composition of the cell wall of the Emerald and Jewel cultivars was analyzed by (HPAEC-PAD) to better understand whether the differences observed in firmness between these two cultivars could be related to differences in the monosaccharide composition of their cell walls (Table S2). To highlight the most relevant differences in the composition of monosaccharides, a comparison was made between harvest and 30 days of cold storage (Figure 2). The data indicate a higher amount of glucose (Glc) detected in the cell wall material obtained from Emerald blueberries (harder cultivar) after 30 d of cold storage. The Glc amount detected by 2M trifluoroacetic acid (TFA) hydrolyzation corresponds mainly to noncellulosic monosaccharides [52]. Additionally, blueberry starch accumulation at harvest is minimal [53,54], suggesting that most of the Glc observed cloud be derived from hemicellulose domains. In relation to galacturonic acid (GalA), no significant differences between cultivars were observed in the cell wall material obtained at both stages (Table S2). However, it should be noted that other modifications, such as methylesterifications, could be modifying the HG status.

### 2.3. Immunohistochemical and Analytical Assays of Emerald and Jewel Blueberries

**Figure 3:**
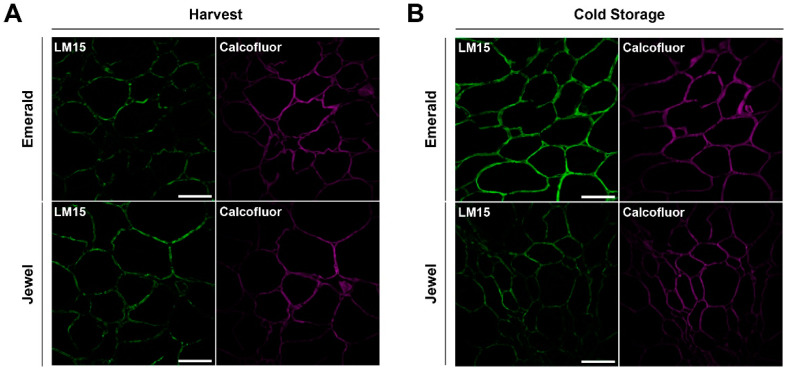
Immunolabeling of blueberry tissues from “Emerald” and “Jewel” cultivars at harvest and cold storage. Xyloglucan detection (green) at harvest (**A**) and cold storage (**B**). Counterstaining of cellulose (magenta). Bars = 100 µm (n = 3).

Based on the observation that a significant difference in cell wall monosaccharide composition was detected among blueberry cultivars with different firmness, we analyzed probable differences in cell wall structure in muro. An immunohistochemical assay was carried out using antibodies with different affinities for cell wall polymers (Material and Methods section). The images obtained from confocal microscopy showed that cell walls from Emerald blueberries displayed a similar fluorescent signal than did the cell walls of Jewel samples at harvest when probed with the antibody that binds to xyloglucan domains (XXXG motif, LM15 [Figure 3A]). Notwithstanding, during cold storage, an increased LM15 fluorescent signal was detected in Emerald cell walls than Jewel phenotype (Figure 3B), and this correlates with the higher concentration of Glc detected by HPAEC-PAD analysis of the Emerald cultivar (Figure 2). These findings suggest that xyloglucan dynamics could be relevant to blueberry firmness.

### 2.4. Firmer Blueberries Are Associated with HG Methylesterification Status and a Higher Cell Wall Calcium Content

**Figure 4:**
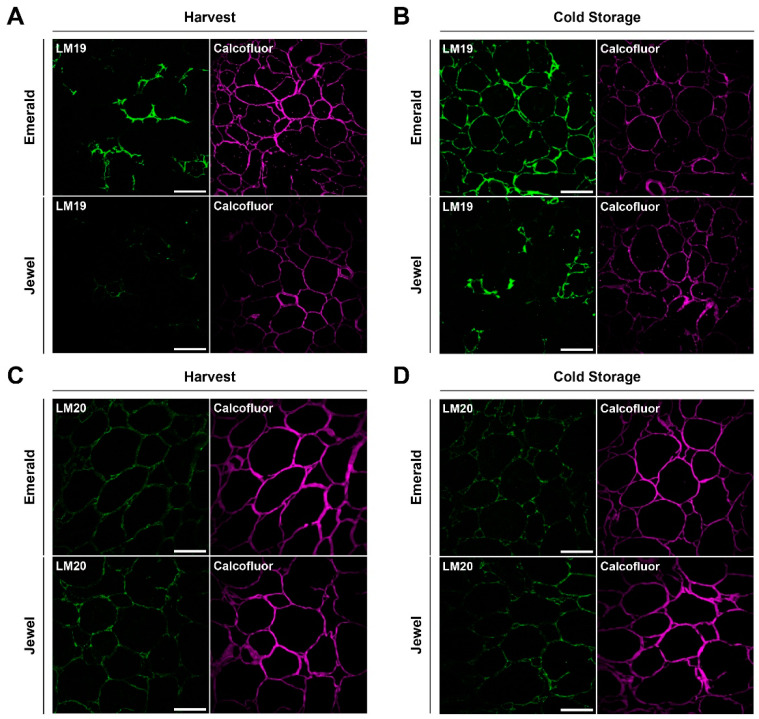
Immunolabeling of blueberry tissues from “Emerald” and “Jewel” cultivars at harvest and after 30 d of cold storage. Demethylesterified homogalacturonan detection (green) at harvest (**A**) and cold storage (**B**). Highly methylesterified homogalacturonan detection (green) at harvest (**C**) and after 30 d of cold storage (**D**). Counterstaining of cellulose (magenta). Bars = 100 µm (n = 3).

Since HG is the pectin most strongly associated with fruit firmness in different fruit models [55,56,57], all samples were probed with LM19 and LM20 antibodies. These probes are able to bind HG with different modifications, such as demethylesterified and highly methylesterified domains, respectively. Figure 4A shows the binding of the LM19 antibody to HG, indicating that the firmer phenotype (Emerald) exhibits a higher fluorescent signal than does the softer phenotype (Jewel). The same fluorescent pattern was detected between both phenotypes after the 30 d cold storage period (Figure 4B). Additionally, the demethylesterification level of HG increased for both phenotypes following the cold storage period relative to the harvest period (Figure 4A,B). Furthermore, Figure 4C,D show the binding of the LM20 antibody to the highly methylesterified HG, and no differences were observed in the cell walls of both cultivars at harvest and after 30 d of cold storage (Figure 4C,D). These results suggest that the firmness of blueberries is associated with the degree of HG methylesterification during harvest and cold storage.

**Figure 5:**
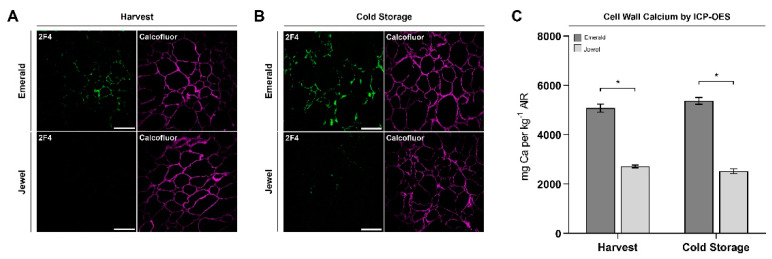
Immunolabeling of blueberry tissues from “Emerald” and “Jewel” cultivars at harvest and after 30 d of cold storage. Homogalacturonan/calcium dimer detection (green) at harvest (**A**) and after cold storage (**B**). Counterstaining of cellulose (magenta). Bars = 100 µm (n = 3). Cell wall calcium quantification of the Emerald and Jewel cultivars at harvest and after 30 d of cold storage (**C**). Error bars represent SEM (n = 5). Data were analyzed by *t*-test (* *p* < 0.05).

In order to understand HG dynamics during harvest and cold storage in firm and soft blueberries, we considered whether the HG epitopes could be bound to calcium ions forming an eggbox motif contributing to blueberry firmness. For this reason, the 2F4 antibody, which binds to a dimeric association of HG chains through calcium ions, was probed. Figure 5A indicates that the cell walls of the Emerald blueberries exhibited a stronger fluorescent signal at harvest stage, whereas the Jewel blueberries displayed a weakly visible fluorescent signal. A similar fluorescent pattern was observed after 30 d of cold storage (Figure 5B). Moreover, cell wall-associated calcium increases were found in both phenotypes after 30 d of cold storage relative to at harvest time (Figure 5A,B). 

The observation that cell walls from the Emerald blueberries exhibited a stronger fluorescent signal that did the Jewel blueberries when probed with the 2F4 antibody suggests that calcium could play a role in blueberry firmness at both timepoints. Since calcium should be associated with cell wall material to participate in this process, we analyzed the calcium concentration present in the cell wall material (alcohol-insoluble residue, AIRs) obtained from blueberries of Jewel and Emerald cultivars at harvest and after 30 d of cold storage. Interestingly, more calcium was found in the cell wall material isolated from Emerald blueberries at both timepoints compared to Jewel samples (Figure 5C). This observation supports the idea that cell wall-associated calcium could play a role in blueberry firmness in these cultivars.

## 3. Discussion

This work revealed that an increase in neutral sugars, such as glucose, is associated with a higher fruit firmness in the Emerald cultivar as compared to the softer Jewel blueberries after 30 d of cold storage. These results also indicate that there are detectable changes in cell wall-associated calcium between both cultivars. These observations expand the scope of the evidence in support of the hypothesis that hemicelluloses metabolism and cell wall calcium play an important role in blueberry firmness postharvest.

### 3.1. Increased Xyloglucan Amount Is Associated with Blueberry Firmness during Cold Storage

The results obtained from the analysis of cell wall components, as well as the use of antibodies, would point to an important role of the hemicellulose metabolism, and especially of xyloglucans, in the textural characteristics of blueberries postharvest. Different researchers have pointed out that during fruit development and ripening, hemicelluloses undergoes modifications that have an impact on the textural characteristics, which would help to maintain the stability of the cell wall structure and firmness of the fruit [16,17,58,59]. In the case of blueberry, it has been determined that modifications of hemicelluloses would be of particular importance due to the large amount of this type of biopolymer present in the cell wall of these fruits [49,50,60], which coincides with the results of the present work. Likewise, of special interest are the modifications that not only occur in fruit growth, but also postharvest. In this regard, the researchers studied the metabolism of the hemicelluloses of pears in refrigerated storage, determining that in the level of changes correlated with the extension of time in storage [61]. Similarly, kiwifruit with different softening rates were compared, and it was found that the slow softening genotype showed higher concentrations of glucose and xylose, hemicellulose associated components, in the later stages of softening [62]. This aspect is relevant, given that in cold storage the cell wall continues to undergo modifications that affect fruit firmness. This situation of wall remodeling postharvest has been analyzed using apples as a study model [63]. These researchers determined that during refrigerated storage genes associated with both cell wall synthesis and remodeling were actively transcribed and found marked differences between firm versus soft firmness genotypes. Similarly, a study conducted to understand the changes of Honeycrisp apples, which is characteristic to present a significant firmness postharvest, found that this could be explained in part by the ability to continue cell wall synthesis in cold storage [64]. In addition, these authors determined that genes associated with hemicellulose metabolism, such as xyloglucan endotransglycosylase/Hydrolase (XTH), may be particularly involved in maintaining apple firmness after harvest. In this aspect it is very noteworthy that the experiments where tomato XTH was overexpressed [65] obtained a lower depolymerization of the xyloglucans that were correlated with a higher fruit firmness, suggesting that XTH is relevant in the maintenance of cell wall stability. Similarly, the investigators overexpressed an endotransglucosylase/xyloglucan hydrolase (XTH1) obtained from persimmons in tomato, achieving significantly reduced fruit softening [66]. Thus, and based on the information present in the literature, and in agreement with the data obtained in the present work, the metabolism of hemicelluloses, and in particular of xyloglucan, would have a great impact on the firmness of the blueberry fruits postharvest. 

### 3.2. Homogalacturonan Methylesterification Status and Calcium Dynamics Are Correlated to Blueberry Firmness

**Figure 6:**
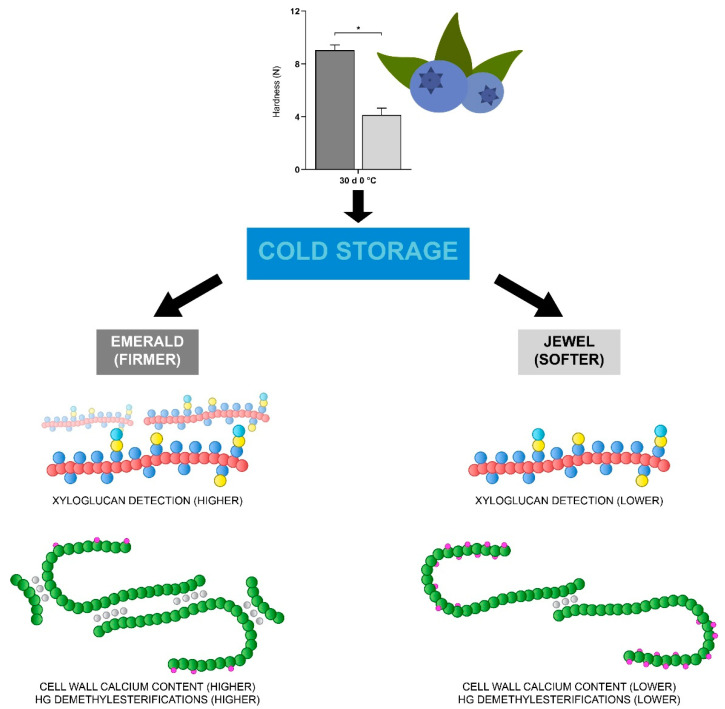
Schematic representation of main results showing xyloglucan detection (red backbone) and cell wall-associated calcium and methylesterification status of GalA (green backbone) for Emerald and Jewel cultivars (* *p* < 0.05).

Pectins are the cell wall component that has been most associated with the firmness of the fruit [67,68]. The HGs are the most abundant constituent of pectins, and their methylesterification dynamics allows for remodeling that has been correlated positively and negatively with the firmness of various fruit [57]. Recently, a proteomic and metabolomic characterization of blueberries with contrasting quality parameters indicated that at ripening stage, an intense cell wall recycling is described in the Emerald cultivar, suggesting that such recycling may involve pectin metabolism and could contribute to the maintenance of a firmer phenotype [69]. The results obtained in this work suggest that the chains of HG contain a higher number of demethylesterified regions in firmer blueberries, and highly methylesterified regions are present in both blueberry phenotypes in similar proportions, as assayed using LM19 and LM20 antibodies, respectively. The removal of methylesterifications are mediated by the enzyme pectin methylesterase (PME; [70]). The PME activity in blueberries has been described in contrasting firmness phenotypes, indicating that firmer cultivars show a slightly decreased PME activity at ripening compared to a softer cultivar [10]; however, the firmer phenotype used in this work exhibited a higher LM19 fluorescent signal than the softer phenotype. Demethylesterified HG chains can undergo two fates: enzymatic degradation or binding to calcium ions. Due to the similar fluorescent patterns of LM20 antibody between both phenotypes, and a lack of difference between the galacturonic acid (GalA) amounts detected by HPAEC-PAD, we suggest that methylesterification status is associated with the cell wall calcium dynamics. An interesting observation was the detection of HG-associated calcium by the 2F4 antibody, in addition to the detection of a higher amount of calcium in cell wall material derived from blueberries of the Emerald cultivar at harvest and after cold storage compared to Jewel blueberries. Since Emerald blueberries exhibit a higher firmness, it is tempting to speculate that cell wall-associated calcium plays a role on blueberry firmness. Previous work by other authors suggests that supplementing the soil with calcium during the previous season enhances fruit firmness at harvest and postharvest [14], and the foliar application of fertilizers containing calcium chloride helps to achieve higher firmness and resistance to mechanical damage in “Duke” cultivars [46]. Furthermore, for the “Elliot” cultivar, preharvest foliar applications helped to increase fruit texture at postharvest stages [38], suggesting that an increase in the amount of unattached calcium in the plant and fruit cloud allow a greater maintenance of firmness. Based on the information obtained, the following scheme is proposed (Figure 6), which summarizes the main results found in this research. In sum, these reports highlight the importance of the cell wall calcium on fruit firmness and warrant additional research to better understand and delineate the role of this ion in this fruit parameter [71].

## 4. Materials and Methods

### 4.1. Plant Material and Phenotypic Analysis

Blueberries (Vaccinium corymbosum) of two cultivars “Emerald” and “Jewel” were harvested during the 2016–2017 season. The fruits were collected from 10 homogeneous bushes per cultivar, following the harvest index of 90% blue fruit color obtained at peak harvest. The trial was conducted in a commercial orchard located in Ovalle (30°39′58.6″ S, 71°04′59.2″ W; 300 m.a.s.l), Coquimbo Region, Chile. The bushes were planted on a sandy loam soil, and water was supplied by drip irrigation system. Orchard management of fertilization, pruning, and pest control was realized according standard procedures for this crop in Chile. Immediately after harvest, fruits were cooled and transported under refrigerated conditions (5 °C and 85% RH) to the laboratory facilities. Fruits similar in weight and color and without external defects were selected for the study. Within each cultivar, berries were selected for characterizing maturity stage by measuring berry weight, diameter, total soluble solids (TSS, %), and titratable acidity (TA, % citric acid). A cold storage assay was carried out after 30 d stored at 0 °C, and the relative humidity was kept above 90% to prevent excessive water loss.

### 4.2. Texture Analysis

Fruit firmness was assessed using the TA.XT Plus Texture Analyser (Stable Micro Systems Ltd., Godalming, UK) provided a texture profile analysis (TPA; Table S1), as described in [6], with some modifications. Briefly, a 5 kg load cell and a flat surface probe (2.5 × 2.5 cm) were used in the compression test. The force was measured on the sagittal side of fruit (n = 10), with the following instrumental settings: test speed of 48* mm/min, post-test speed of 300 mm/min, auto force trigger of 5 g and stop plot at target position. Each berry was compressed twice until deformation of 30%, and the data were acquired with a resolution of 500 pps (points per second).

### 4.3. Determining Cell Wall Monosaccharide Composition

The alcohol-insoluble residues (AIRs) were prepared according to [72], with some modifications. Briefly, 8 g of a pooled frozen blueberries (n = 20) were ground and boiled in 50 mL of 95% (*v*/*v*) ethanol for 10 min. The samples were then centrifuged at 3000× *g* for 15 min, and the supernatant was discarded; this step was performed a total of three times. The pellets were washed using 100% (*v*/*v*) acetone, and the cell wall solid materials obtained were dried overnight at 40 °C.

AIR hydrolyzation and monosaccharide composition were also determined using the same high-performance anion-exchange chromatography with pulsed amperometric detection (HPAEC-PAD) conditions described by [72]. Briefly, 2 mg of AIRs were hydrolyzed with 400 μL of 2 M trifluoroacetic acid (TFA) for 1 h at 121 °C. Then, the TFA was evaporated using gaseous nitrogen at 65 °C for 30 min, and the dried pellets were washed twice with 300 μL of 100% (*v*/*v*) isopropanol. The hydrolyzed AIRs were resuspended in 1 mL of Milli-Q water and filtered through a 0.45-μm syringe filter. The suspension was injected into a HPAEC-PAD (Dionex DX-600 Ion Chromatography System). For sugar quantification, we used two serially connected CarboPac PA1 (4 mm × 250 mm) analytical columns and a CarboPac PA1 (4 mm × 50 mm) guard column. Sugars were separated at 26 °C with a flow rate of 1 mL per min. The elution protocol was conducted by isocratic gradients of 20 mM NaOH for 20 min, followed by 150 mM sodium acetate in 100 mM NaOH for 15 min, and a washing step with 200 mM NaOH for 10 min. A standard curve was used as reference to determine sugar content, which was expressed as g sugar per kg^−1^ AIR.

### 4.4. Immunohistochemistry Assay

Samples from the Emerald and Jewel blueberry cultivars were fixed in a solution of FAA (formaldehyde–acetic acid–ethanol) at a dilution of 2:1:10. The samples were dehydrated using a slow dehydration method consisting of a battery of ethanol, xylol, and Paraplast^®^ (Sigma-Aldrich, St. Louis, MO, USA), as described in Table S3. The samples were then embedded in Paraplast^®^ using inclusion molds and cryosectioned into 3-µm slices with a Leica Jung RM2035 Microtome (Leica Biosystems, Wetzlar, Germany). Three sectioned replicates per sample were performed for subsequent immunohistochemical analyses. The immunohistochemistry assay was modified from [73]. Briefly, samples were treated with xylol for 30 min, followed by chloroform treatment for 30 min. A battery consisting of ethanol/1X PBS (phosphate-buffered saline) was used to rehydrate the samples by submersion, as described in Table S4. The samples were submerged in 1X PBS for 5 min and then pretreated with 0.4% (*v*/*v*) Triton X-100 in 1X PBS for 2 h. Samples were blocked using 5% (*w*/*v*) milk protein in 1X PBS for 2 h.

The primary antibodies used for the recognition of different regions of the cell wall are described in Table S5 (LM15 [74]; LM19 and LM20 [75]; 2F4 [76]). The secondary antibodies used were antirat IgG, antirat IgM, or antimouse IgG conjugated to Alexa 488 (Thermo Fisher Scientific, Rockford, IL, USA) at a concentration of 1:500 diluted in 1X PBS. Calcofluor white M2R (Sigma-Aldrich, St. Louis, MO, USA) was used for counterstaining at a concentration of 50 mg L^−1^ diluted in 1X PBS. Samples were treated as follows: an incubation in 1X PBS for 5 min to remove excess milk protein, followed by an incubation with the primary antibody overnight. Then, the samples were washed three times using 1X PBS for 5 min each and incubated with the secondary antibody for 2 h, and samples were washed three times with 1X PBS for 5 min each. Finally, the samples were incubated using calcofluor white M2R for 10 min, washed three times with 1X PBS, and mounted with Neo-Mount^®^ (Sigma-Aldrich, St. Louis, MO, USA). Confocal images were obtained with a Leica LSI confocal microscope (Leica Microsystems, Wetzlar, Germany), using an optical zoom of 3.6 and a digital zoom factor of 1.0. Images were processed using the software Leica Application Suite X (LASX) version 3.4.2 from Leica Microsystems (Wetzlar, Germany).

### 4.5. Quantification of Cell Wall Calcium

The calcium concentration in the cell wall was determined as described by [77] using 100 mg of blueberry AIRs, obtained in a manner similar to that described in point 4.3., via inductively coupled plasma optical emission spectrometry (ICP-OES). Results were expressed as mg of calcium per kg^−1^ AIR.

### 4.6. Statistical Analyses

T-test was performed using R software version 4.0.2 (Vienna, Austria) at *p* < 0.05. Experiments were carried out using at least three biological replicates.

## 5. Conclusions

Analytical assays showed that firmer blueberries contain an increased amount of xyloglucans and demethylesterified HG. In addition, a higher concentration of cell wall-associated calcium was found in the firmer blueberries, suggesting that there is a pectin-calcium and hemicellulose dynamic associated with contrasting firmness in blueberries at harvest and during cold storage.

## Data Availability

Data sharing not applicable.

## Supplementary Materials

The following are available online at https://www.mdpi.com/2223-7747/10/3/553/s1, Table S1: Phenotyping parameters in blueberry cultivars at harvest and after cold storage stages; Table S2: Cell wall monosaccharide composition (g Kg^−1^ AIR) at harvest and during cold storage for Emerald (hard) and Jewel (soft) blueberry phenotypes; Table S3: Procedure for dehydrating and infiltrating tissues; Table S4: Procedure for rehydrating embedded sections; Table S5: Antibodies used to immunolabel plant cell wall polysaccharides.
