# Supplementary material for: Cell Wall Calcium and Hemicellulose Have a Role in the Fruit Firmness during Storage of Blueberry (Vaccinium spp.)
Source: Plants (Basel). 2021 Mar 16;10(3):553. doi: 10.3390/plants10030553 (PMC7999294; doi:10.3390/plants10030553)
Supplement: Supplementary file 1 [file plants-10-00553-s001.pdf]

Supplementary Table 1. Phenotyping parameters in blueberries cultivars at harvest and after cold storage stages.

	Stage	Weight (g)	Diameter (mm)	Total Soluble Solids (%)	Titratable Acidity (%)	Hardness (N)	Cohesiveness	Chewiness (mJ)	Springiness (mm)	Gumminess (N)	Resilience
Emerald	Harvest	2.09 *A*	16.12 *A*	13.00 *A*	0.48 *B*	10.82 *A*	0.33 *A*	11.44 *A*	3.22 *A*	3.56 *A*	0.14 *A*
Jewel		1.66 *A*	14.58 *B*	11.10 *B*	0.54 *A*	5.24 *B*	0.31 B	4.70 *B*	2.86 *B*	1.64 *B*	0.12 *B*
Emerald	Cold Storage	-	-	13.50 *a*	0.38 *a*	8.56 *a*	0.40 *a*	10.02 *a*	2.99 *a*	3.37 *a*	0.17 *a*
Jewel		-	-	11.00 *b*	0.45 *a*	5.07 *b*	0.40 *a*	6.13 *b*	3.18 *a*	1.92 *b*	0.16 *a*

Different letters within a column indicate significant differences between blueberry phenotypes at each stage using a t-test ($p < 0.05$).
Upper-case letters stand for harvest comparison and lower-case letters stand for cold storage comparison between cultivars.

Supplementary Table 2. Cell wall monosaccharide composition (g Kg^{-1} AIR) at harvest and during cold storage for Emerald (hard) and Jewel (soft) blueberry phenotypes.

	Stage	Rha	Ara	Gal	Glc	Man + Xyl	GalA	GlcA
Emerald (firmer)	Harvest	9.11aA	37.53bA	44.83bA	25.95aB	145.09aA	75.04aA	2.38aA
	Cold storage	5.71aB	38.28bA	50.14bA	79.14aA	123.89aA	58.06aA	2.35aA
Jewel (softer)	Harvest	8.14aA	53.24aB	66.82aA	28.88aA	127.01aA	79.52aA	2.03aA
	Cold storage	6.33aB	58.79aA	60.65aB	39.53bA	83.34bB	53.90aB	1.76bA

Different lower-case letters stand for significant differences between blueberry phenotypes at each stage and, upper-case letters stand for significant differences between stages within same phenotype (n = 6). Data were analyzed by t-test (*$p < 0.05$).

Supplementary Table 3. Procedure for dehydrating and infiltrating tissues.

Step	Solution	Incubation time	Temperature
1	10% (v/v) ethanol/dH_20	60 min	4 °C
2	30% (v/v) ethanol/dH_20	60 min	4 °C
3	50% (v/v) ethanol/dH_20	120 min	4 °C
4	70% (v/v) ethanol/dH_20	120 min	4 °C
5	85% (v/v) ethanol/dH_20	120 min	4 °C
6	95% (v/v) ethanol/dH_20	Overnight	4 °C
7	100% ethanol	120 min	4 °C
8	30% (v/v) xylol/ethanol	120 min	4 °C
9	50% (v/v) xylol/ethanol	120 min	4 °C
10	70% (v/v) xylol/ethanol	120 min	4 °C
11	100% xylol	60 min	RT
12	70% (v/v) xylol/Paraplast®	60 min	60 °C
13	40% (v/v) xylol/Paraplast®	60 min	60 °C
14	100% Paraplast®	Overnight	60 °C
15	100% Paraplast®	48 h	60 °C

RT: Room temperature; dH_2O: distilled water.

Supplementary Table 4. Procedure for rehydrating embedded sections.

Step	Solution	Incubation time	Temperature
1	100% ethanol	5 min (X2)	RT
2	95% ethanol/1X PBS	5 min (X2)	RT
3	80% ethanol/1X PBS	5 min	RT
4	70% ethanol/1X PBS	5 min (X2)	RT

RT: Room temperature; PBS: phosphate buffered saline.

Supplementary Table 5. Antibodies used to immunolabel plant cell wall polysaccharides.

Antibody	Specificity	Dilution	Category
LM15	Anti-(XXXG)-xyloglucan	1:50	Monoclonal
LM19	Anti-un-esterified homogalacturonan	1:50	Monoclonal
LM20	Anti-esterified homogalacturonan	1:50	Monoclonal
2F4	Anti-homogalacturonan/calcium dimer	1:50	Monoclonal
